# Occurrence of congenital disorders in Swiss sheep

**DOI:** 10.1186/1751-0147-55-27

**Published:** 2013-03-22

**Authors:** Deborah Greber, Marcus Doherr, Cord Drögemüller, Adrian Steiner

**Affiliations:** 1 Clinic for Ruminants, Vetsuisse Faculty, University of Bern, Bremgartenstrasse 109a, Bern, 3001, Switzerland; 2 Veterinary Public Health Institute, Vetsuisse Faculty, University of Bern, Schwarzenburgstrasse 155, Liebefeld, 3097, Switzerland; 3 Institute of Genetics, Vetsuisse Faculty, University of Bern, Bremgartenstrasse 109a, Bern, 3001, Switzerland

**Keywords:** Genetic disorders, Congenital disease, Sheep, Entropion, Brachygnathia inferior, Hernia, Cryptorchidism, torticollis

## Abstract

**Background:**

The rates of congenital disorders in Swiss sheep were determined by a questionnaire which was sent to 3,183 members of the Swiss Sheep Breeders’ Association.

**Findings:**

A total of 993 questionnaires were returned, giving a response rate of 31.2%. Of these, 862 questionnaires originated from farms keeping one of the predominant Swiss sheep breeds: Swiss White Alpine sheep, Brown-Headed Meat sheep, Swiss Black Brown Mountain sheep and Valais Blacknose sheep. During a 10-year-period, entropion was reported in 33.6% of the farms, brachygnathia inferior in 29.5%, abdominal/umbilical hernia in 15.9%, cryptorchidism in 10.5% and torticollis in 10.5%. The most significant difference between the four breeds (*P* < 0.001) occurred for entropion in Swiss White Alpine sheep and Brown-Headed Meat sheep, brachygnathia inferior in Swiss Black Brown Mountain sheep, and scrotal/inguinal hernia in Valais Blacknose sheep. The Swiss White Alpine breed showed a significantly higher animal prevalence of entropion (6.2% in 2011 and 5.5% in 2012) than other breeds (*P* < 0.001).

**Conclusions:**

These findings indicate a breed-specific necessity for action, particularly regarding Swiss animal welfare legislation, especially entropion in Swiss White Alpine sheep is concerned. In general, careful selection of breeding stock is to be recommended.

## Background

Modern sheep production is primarily focused on economically important traits like meat and milk. The productivity of certain breeds has improved remarkably due to efficient breeding programs rooted in quantitative genetics. However, although stringent selection increases profitability, it may reduce effective population size, particularly when single elite rams and their progeny are used intensively. The accompanying increase in co-ancestry and inbreeding leads to the emergence of recessive genetic defects, which may cause significant economic and animal welfare concerns. For instance, in the last decade, outbreaks of arthrogryposis were reported in Suffolk sheep [1], microphthalmia in Texel sheep [2], epidermolysis bullosa in German Black-Headed Mutton [3], inherited rickets in Corriedale sheep [4], and cervical vertebral malformation in Texel sheep [5]. Long before the era of quantitative genetics such defects also emerged from time to time due to the limited availability of unrelated rams. Breeders have always tended to mate related individuals, partly due to a lack of unrelated animals, and partly in order to breed phenotypically similar animals. Nowadays this still holds true, especially in small populations e.g. of endangered breeds. It is crucial that sheep breeders implement effective strategies to control in-breeding and subsequent increased numbers of defects. A path towards that goal is to establish surveillance to detect emerging defects and collect DNA samples of defective animals. Such material can be used to study the underlying genetics of recorded defects and to develop gene tests for selection.

In the past, the occurrence of undesirable traits recognized as inherited disorders in sheep has been reported [5-8]. However, a significant number of unrecorded cases is likely to have occurred have probably remained unreported [7]. Congenital disorders are not necessarily inherited as they may be due to environmental or infectious factors [7,9,10]. However, it is known that many congenital disorders have a genetic cause. Some of the common genetic disorders in sheep are atresia ani, arthrogryposis, brachygnathia, chondrodysplasia, cleft palate, cryptorchidism, entropion, hernia, and torticollis [5,6]. A continuously updated database reports a total of 215 inherited ovine defects, disorders, predispositions to disease, production traits and coat colors [11]. A recent review gives an overview of predominantly recessive inherited ovine genetic traits, for which causal gene variants are known and where gene testing can be used to eliminate these undesirable mutations [12].

It is well known that the prevalence of congenital disorders varies among breeds and among lines within breeds. Once recognized, they may not be widely reported by breeders because of image damage and the financial consequences of not being able to sell potentially valuable stock. Nowadays, animal protection and welfare are of increasing importance to sheep breeders as well as to consumers. The Swiss Protection of Animals Act [13] and the Swiss Protection of Animals Ordinance [14] define that only healthy animals should be used for breeding.

**Figure 1:**
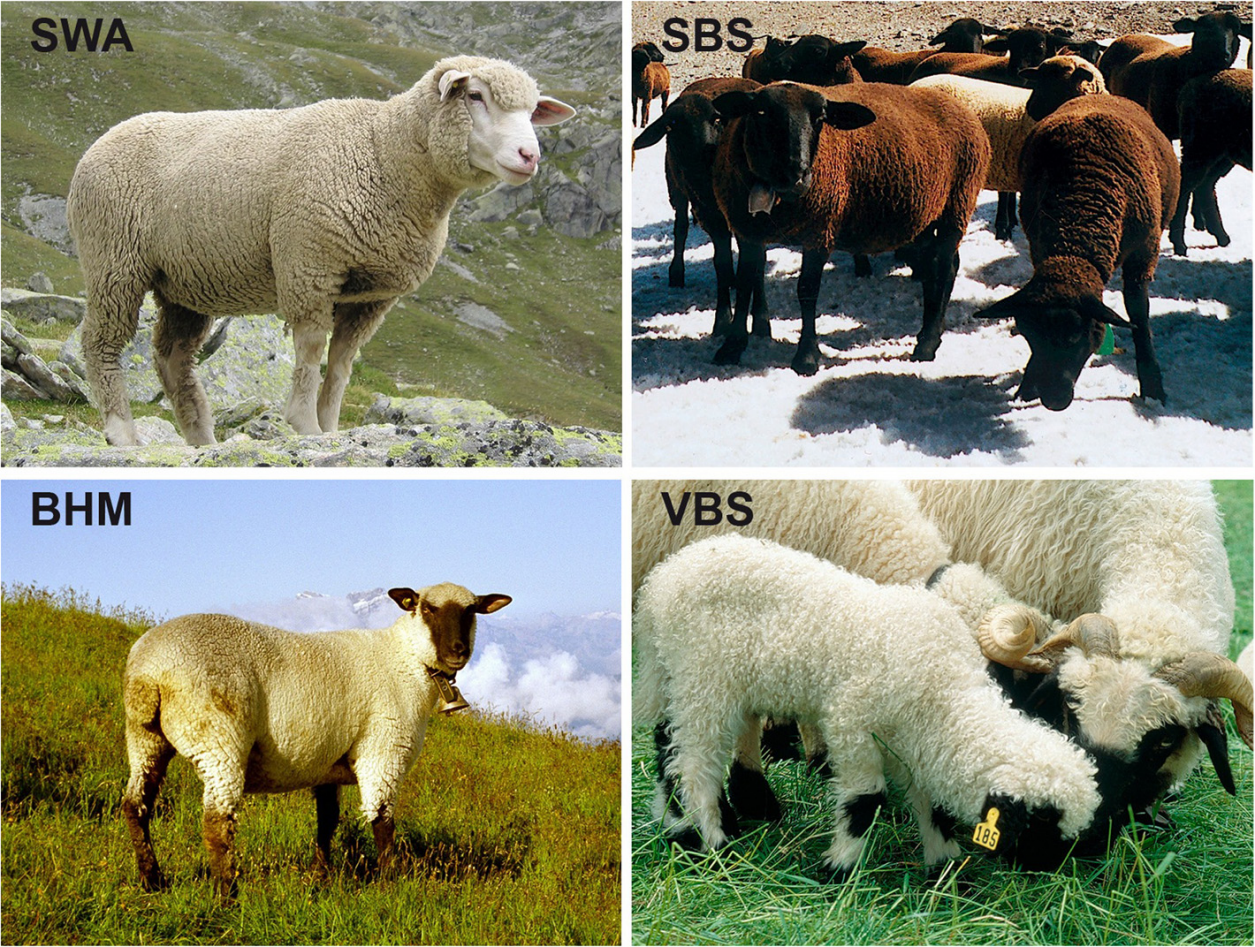
**Representative breed individuals.** (SWA = Swiss White Alpine sheep; BHM = Brown Headed Meat sheep; SBS = Swiss Black Brown Mountain sheep; VBS = Valais Blacknose sheep). Pictures were provided by the Swiss Sheep Breeders’ Association.

Switzerland has several indigenous and locally developed breeds of sheep with unique combinations of adaptive traits to best respond to local environment pressures. In addition, several imported breeds are also established as synthetic breeds after introgression into local breeds. Presently, about 20% of Swiss sheep are registered in a herd book. The aim of this study was to describe the occurrence of congenital diseases and defects in the four main Swiss breeds of sheep, comprising more than 10,000 registered breeding animals. Therefore, we performed a survey in two certified native breeds, the Swiss Black Brown Mountain sheep (SBS) and the Valais Blacknose sheep (VBS), and two synthetic breeds, the Swiss White Alpine sheep (SWA) and the Brown-Headed Meat sheep (BHM) (Figure 1).

The SBS breed was mentioned as far back as the 14th century and is a composite of different land races. Originally, it was bred for its dark, fine, high quality wool. The breed only became standardized at the beginning of the 20th century and today it accounts for about 15% of the total Swiss sheep registered. The VBS breed was first mentioned in the 15th century and today accounts for about 17% of the total sheep registered. This breed boasts exceptional characteristics which have been acquired through centuries, such as adaptability to the harsh mountain climate, good conversion of roughage, fecundity, an affable nature and loyalty to its home range.

In addition, the two predominant synthetic Swiss sheep breeds were included: SWA constitutes about 50% of total sheep registered and is a composite of several mountain breeds. Ongoing cross-breeding with foreign breeds like Merino, Texel and Ile de France has sustainably improved the meat and wool yield of the original land race sheep. The BHM breed represents one of the heaviest sheep in Switzerland and accounts for about 17% of the total sheep registered. It is the result of crossbreeding the English Oxford Downs and the German Black-Headed Mutton with the native Grabs sheep which was common to Switzerland in the 19th century. Crossbreeding with these foreign natives is still permitted to improve the breed.

This study presents the results of a questionnaire on congenital disorders sent to all members of the Swiss sheep breeders’ association and attempts to estimate the occurrence of congenital disorders in the four main Swiss sheep breeds.

## Materials and methods

The sheep owner agreed that the data from the questionnaire can be used for our study. All animal information has been obtained according to the national and international guidelines for animal welfare. There was no “animal experiment” according to the legal definitions in Switzerland.

As a first step, a questionnaire was sent to members of the Swiss sheep breeders’ association that represents the herd book breeders of the four main sheep breeds included in this study. Before the questionnaire was sent to the breeders, they were informed about the common congenital disorders in a short article in a journal sent to all breeders and by oral presentations. In total, 3,183 questionnaires were sent to the breeders in early April 2012. The deadline for answers and return of the questionnaire was the 5th May 2012. As an alternative, a link to an internet-based version of the questionnaire was also provided. All answers received by the 13th May 2012 were included in the results.

The questionnaire consisted of two parts. In the first part, questions on the main breed for each flock were given: the numbers of ewes and rams on the farm at April 2012, the number of lambs in a 12-month period from January to December 2011 and in a 4-month period from January to April 2012. The breeders were also asked to report if one of the following defects had occurred during the last ten years: arthrogryposis, atresia ani, brachygnathia inferior, abdominal/umbilical hernia, scrotal/inguinal hernia, cleft palate, cryptorchidism, entropion, hydrocephalus, microphthalmia, and torticollis. We grouped the four types of hernia into two groups: abdominal/umbilical hernia and scrotal/inguinal hernia, respectively, because it may not be possible for breeders to distinguish between hernia with an almost similar appearance. In the second part, which focused on entropion, we asked for the number of affected lambs with this defect from January 2011 to December 2011 and from January to April 2012.

The results were analyzed using the statistical program NCSS 2007. The statistical significance of the difference between two independent proportions was tested with Fisher’s exact and chi-square test. The *P*-value for a statistically significant difference was set at < 0.05.

## Results and discussion

We sent questionnaires to the 3,183 members of the Swiss sheep breeders’ association, which represents about a third of all 9,428 registered sheep farmers in Switzerland in 2011 [15]. A total of 993 questionnaires were returned (31.2%), 862 of which dealt with one or more of the four breeds included: 385 flocks of SWA, 204 of BHM, 150 of SBS, and 123 of VBS, respectively. The 862 farmers corresponded to 9.1% of all sheep farmers in Switzerland.

In 2011, 68,165 animals of the four main Swiss breeds were registered in the herd book while totally 424,018 sheep were registered in the Swiss Stock Movement Database in 2011 [15]. A total of 19,982 sheep were included in the study in 2011 corresponding to 4.7% of the total registered Swiss sheep population.

As in our survey, the questionnaires were only being sent to herd book members. It can reasonably be concluded that the results tend to be at the lower limit. Herd book breeders might pay more attention to potentially congenital disorders and exclude potentially affected lambs from breeding than non-members.

### Occurrence of congenital disorders

**Figure 2:**
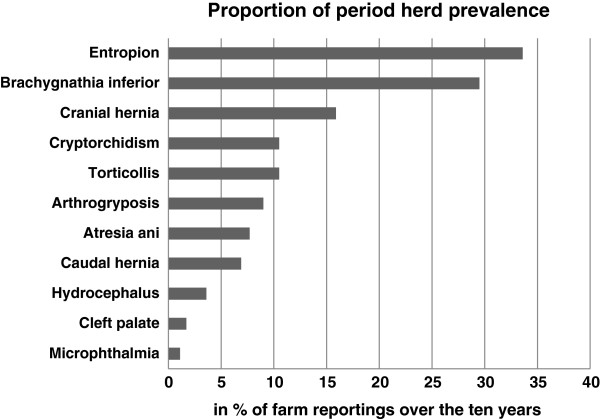
Proportion of farm reporting of congenital disorders of the four main Swiss sheep breeds, over a 10-year-period (2002–2012).

The proportion of farms reporting congenital disorders across the four breeds from 2002–2012 is shown in Figure 2. At farm level, the most prevalent defects were: entropion 33.6%, brachygnathia inferior 29.5%, abdominal/umbilical hernia 15.9%, cryptorchidism 10.5%, torticollis 10.5%, arthrogryposis 9%, atresia ani 7.7%, scrotal/inguinal hernia 6.9%, hydrocephalus 3.6%, cleft palate 1.7%, and microphthalmia 1.1%. Statistical analyses of the frequency of cleft palate, arthrogryposis, microphthalmia, and torticollis, showed insignificant differences among the four breeds, while significant differences were found for entropion, brachygnathia inferior, and scrotal/inguinal hernia (*P* <0.001) (Table 1).

**Table 1:** Data on farm reporting congenital disorders in four Swiss sheep breeds during a 10-year-period (2002–2012)

		**SWA**	**BHM**	**SBS**	**VBS**	**Total**	**z**^**2**^P*
**Arthrogryposis**	No. of farm reports	43	14	13	6	76	0.12
	Total no. of farms	380	200	148	119	847	
	Prevalence (%)	11.3	7	8.8	5	9	
**Atresia ani**	No. of farm reports	42	11	7	5	65	0.01
	Total no. of farms	380	199	148	119	846	
	Prevalence (%)	11.1	5.5	4.7	4.2	7.7	
**Brachygnathia**	No. of farm reports	131	34	66	19	250	<0.001
**inferior**	Total no. of farms	381	199	148	119	847	
	Prevalence (%)	34.4	17.1	44.6	16	29.5	
**Cleft palate**	No. of farm reports	8	1	4	1	14	0.31
	Total no. of farms	381	199	148	119	847	
	Prevalence (%)	2.1	0.5	2.7	0.8	1.7	
**Cryptorchidism**	No. of farm reports	46	23	17	3	89	0.02
	Total no. of farms	379	199	147	119	844	
	Prevalence (%)	12.1	11.6	11.6	2.5	10.5	
**Entropion**	No. of farm reports	202	74	10	3	289	<0.001
	Total no. of farms	385	203	150	123	861	
	Prevalence (%)	52.5	36.5	6.7	2.4	33.6	
**Umbilical/**	No. of farm reports	75	30	22	7	134	0.004
**abdominal hernia**	Total no. of farms	380	198	148	119	845	
	Prevalence (%)	19.7	15.2	14.9	5.9	15.9	
**Scrotal/inguinal**	No. of farm reports	22	4	4	28	58	<0.001
**hernia**	Total no. of farms	380	198	147	119	844	
	Prevalence (%)	5.8	2.0	2.7	23.5	6.9	
**Hydrocephalus**	No. of farm reports	22	4	3	1	30	0.015
	Total no. of farms	379	200	146	119	844	
	Prevalence (%)	5.8	2	2.1	0.8	3.6	
**Microphthalmia**	No. of farm reports	5	3	1	0	9	0.56
	Total no. of farms	381	199	147	119	846	
	Prevalence (%)	1.3	1.5	0.7	0	1.1	
**Torticollis**	No. of farm reports	48	24	9	8	89	0.07
	Total no. of farms	381	198	147	119	845	
	Prevalence (%)	12.6	12.1	6.1	6.7	10.5	

(*SWA* Swiss White Alpine sheep; *BHM* Brown Headed Meat sheep; *SBS* Swiss Black Brown Mountain sheep; *VBS* Valais Blacknose sheep).

*overall *P*-value between breeds.

A previous study on congenital disorders of sheep in Western Australia showed the occurrence of a wide range of defects: bowed forelegs, micrognathia, hermaphroditism, cryptorchidism, prognathia, atresia ani, microtia, entropion, torticollis, polythelia and arthrogryposis in order of decreasing frequency [6]. Our results are not directly comparable with these findings due to different study designs. The Australian survey identified the congenital defects commonly observed in sheep in Western Australia but gave no information on the incidence and cause of the various defects [6]. Our study reports the disorders that the breeders observed from 2002–2012 and provides estimates of the proportion of farms with particular congenital disorders. However, the two studies demonstrate that similar congenital defects occur in both regions.

The significance of the present study is limited by its design as breeders had to remember why they lost lambs in the course of the past ten years. Often, the cause of a single lost lamb remains unnoticed or forgotten while several malformed lambs are more likely to be recorded. It is likely that we have estimated the lower limit of how many farms that actually observed one of these disorders over the last ten years. On the one hand, herd book breeders probably pay more attention to congenital disorders than regular sheep farmers. In fact, we expected fewer questionnaires to be returned. On the other hand, the significant differences between the breeds indicate reliability in answering the questionnaire. We cannot rule out that reporting was biased. The study indicates that Swiss sheep breeders are interested in animal welfare and that breed-specific problems exist, especially entropion, brachygnathia inferior and hernia.

### Entropion

On average, every third flock had cases of entropion during the last 10 years. Significant differences were detected between breeds as SWA had the highest proportion of farms reporting the defect (52.5%), while VBS had the lowest (2.4%). Due to rumors among breeders about a recent occurrence of this genetic defect in newborn lambs, we decided to ask for detailed data on this issue. This enabled us to give an accurate overview of the occurrence of entropion in the last two years (Table 2). The SWA breed showed a significantly higher animal prevalence (6.2% in 2011 and 5.5% in 2012) than in other breeds (*P* < 0.001), while the prevalence of entropion in BHM was significantly higher than in SBS and VBS (*P* < 0.001). There was no significant difference in the prevalence of entropion between SBS and VBS.

**Table 2:** Occurrence of entropion in 2011 (Jan to Dec) and 2012 (Jan to Apr) in the four main Swiss sheep breeds

		**Number of all lambs**	**Lambs affected with entropion**	**Prevalence (%)**	**Proportion (%) of farm reportings of entropion in 2011-2012**
**SWA**	2011	11820	737	6.2	36.9
	2012	7005	385	5.5	
**BHM**	2011	2618	71	2.7	19.2
	2012	2713	66	2.4	
**SBS**	2011	3504	7	0.2	2.7
	2012	2055	7	0.3	
**VBS**	2011	2215	2	0.09	1.6
	2012	1314	5	0.38	

(*SWA* Swiss White Alpine sheep; *BHM* Brown Headed Meat sheep; *SBS* Swiss Black Brown Mountain sheep; *VBS* Valais Blacknose sheep).

Table 2 clearly points out that almost every third farm keeping SWA sheep had lambs affected with entropion in the last two years. In 2011, a total of 737 out of 11,820 lambs were affected with entropion. Therefore, the study confirmed the rumors that the SWA have a higher frequency of lambs with entropion than the other breeds studied. A breed predisposition has not been reported in the literature, but a study has mentioned that entropion occurred more frequently in smaller flocks without regular exchange of the ram than in large populations [16]. Our findings show an urgent need for action against entropion in the SWA breed due to animal welfare reasons. It is advised that animals with affected offspring and the offspring itself should be excluded from breeding. However this may be complicated as successful surgery changes the phenotype as the treated animals become clinically normal. Thus, if the breeder does not accurately keep records on phenotypes, later distinguishing between normal and surgically-treated animals may be impossible. Several breeding experiments have been carried out and Littlejohn [17] claimed that the condition was not inherited as a simple Mendelian recessive defect, but that a genetic disposition existed. Taylor and Catchpole [18] stated that entropion represents an inherited disease. They postulated that the condition is caused by several recessive genes with additive effects. They showed in their experiment that the breed of the ram and the ewe influenced the probability of developing entropion. They bred three purebred Suffolk rams to purebred Scotch Half-bred ewes resulting in 10.8% of lambs with entropion, while the same Suffolk rams set to crossbreed ewes gave an incidence of 1.25% [18]. Comparable to our findings in SWA, a prevalence of 2.7 to 6.6% of entropion in lambs was recorded in three flocks in south west England over a 3 year period [19]. As we have observed in our study these authors showed that the only significant variable associated with the presence of entropion was the breed. Charolais and Texel lambs showed an increased risk of developing entropion when compared to Suffolk lambs [19]. An American study reported an overall mean incidence rate of entropion of 3.1% over a nine-year period, but the prevalence varied from 1.0 to 10.4% between different breeds [20]. Lamprecht and Pfeiffer [16] showed that female lambs are more often affected by entropion than males and observed that through the removal of the fathers of affected lambs, the frequency of the disease could be considerably decreased. Taken together, our data and the published studies on entropion show breed predispositions and indicate likely genetic causes of this disorder. Therefore, selection against this disorder might be performed in future.

### Brachygnathia inferior and hernias

The occurrence of brachygnathia inferior and hernias showed statistically highly significant differences between breeds (Table 1). Brachygnathia inferior was the most common defect (Table 1) and 44.6% of SBS farms had recorded this defect. Brachygnathia inferior is a common ovine defect that may be caused by recessive gene mutations, viruses, phytoteratogens or undetermined factors [7]. In some cases, brachygnathia inferior is presumably a genetic defect with a multifactorial inheritance with several genes being involved [21,22]. A recent breeding experiment showed that brachygnathia inferior in East Frisian milk sheep could not be explained by a simple monogenic Mendelian inheritance [22].

Abdominal/umbilical hernias occurred in equal frequencies in flocks of the SWA, BHM, and SBS breeds. Abdominal/umbilical hernias were significantly less common in VBS flocks (*P* = 0.004) (Table 1). Birth of twins or triplets is more common in the SWA, BHM, and SBS breeds than in the VBS breed, which may affect the observed differences in prevalence among these breeds. Data on this issue was not included in the questionnaire.

Scrotal/inguinal hernia was recorded in a total of 6.9% of flocks in the last ten years. As this defect occurred in a higher proportion in the VBS than in other 3 breeds (*P* < 0.001; Table 1), a breed predisposition can be assumed. Hernias are described to be either congenital or acquired and to occur as isolated defects or associated with defects of other parts of the body [23]. For scrotal hernia it was suggested that the condition has a recessive genetic character [24]. Braun and Cole [25] stated that the inheritance of inguinal and scrotal hernia in the ram is not understood; probably because their data did not fit any simple single-locus model. In the Merino breed, it is assumed, on the basis of a case report, that rams could be genetically predisposed to inguinal hernia [26].

In comparison with the other three breeds, all remaining congenital defects of interest in this survey appeared less frequently in the VBS breed. This result is somewhat surprising, because the VBS breed represents a comparatively smaller population, and it is assumed that they have a narrow gene pool. However, the data seem trustworthy as the reports of elevated numbers of scrotal/inguinal hernia for VBS indicate no concealment of disorders.

## Conclusions

The survey revealed defects occurring at varying rates in four Swiss sheep breeds. There were statistically significant breed predispositions for entropion in the SWA and BHM breed, for brachygnathia inferior in the SBS breed, and for scrotal/inguinal hernia in the VBS breed. The identification of the most common defects might encourage the breeders to eliminate affected animals from their breeding program to decrease the incidence. The efficiency of this could be improved if DNA-based research was undertaken in order to develop genotyping tests.

## Competing interests

The authors declare that they have no competing interests.

## Authors’ contributions

DG, CD and AS conceived and designed the study. DG conducted the survey. DG, CD and AS analyzed the data. DG and MD performed the statistical analysis. DG, CD and AS wrote the paper. All authors read and approved the final manuscript.
